# A Combined Approach of High-Throughput Sequencing and Degradome Analysis Reveals Tissue Specific Expression of MicroRNAs and Their Targets in Cucumber

**DOI:** 10.1371/journal.pone.0033040

**Published:** 2012-03-30

**Authors:** Weihua Mao, Zeyun Li, Xiaojian Xia, Yadan Li, Jingquan Yu

**Affiliations:** 1 Department of Horticulture, Zhejiang University, Hangzhou, China; 2 Center of Analysis and Measurement, Zhejiang University, Hangzhou, China; 3 Hunan Agricultural Bioengineering Research Institute, Hunan Agricultural University, Changsha, China; East Carolina University, United States of America

## Abstract

MicroRNAs (miRNAs) are endogenous small RNAs playing an important regulatory function in plant development and stress responses. Among them, some are evolutionally conserved in plant and others are only expressed in certain species, tissue or developmental stages. Cucumber is among the most important greenhouse species in the world, but only a limited number of miRNAs from cucumber have been identified and the experimental validation of the related miRNA targets is still lacking. In this study, two independent small RNA libraries from cucumber leaves and roots were constructed, respectively, and sequenced with the high-throughput Illumina Solexa system. Based on sequence similarity and hairpin structure prediction, a total of 29 known miRNA families and 2 novel miRNA families containing a total of 64 miRNA were identified. QRT-PCR analysis revealed that some of the cucumber miRNAs were preferentially expressed in certain tissues. With the recently developed ‘high throughput degradome sequencing’ approach, 21 target mRNAs of known miRNAs were identified for the first time in cucumber. These targets were associated with development, reactive oxygen species scavenging, signaling transduction and transcriptional regulation. Our study provides an overview of miRNA expression profile and interaction between miRNA and target, which will help further understanding of the important roles of miRNAs in cucumber plants.

## Introduction

MicroRNAs (miRNAs) are a class of single strand, endogenous, approximately 22 nt non-coding RNA molecules that negatively regulate gene expressions at post-transcriptional level [1], [2]. In plants, the rapidly increasing studies demonstrate that miRNAs play an important role in a broad range of biological processes, including regulation of plant growth development and response to biotic and abiotic stresses via interactions with their specific target mRNAs [3]–[5]. Therefore, numerous efforts have been made to discover and identify miRNAs from diverse plant species in recent years. Initially, the traditional (direct cloning and sequencing) Sanger sequencing and computational predictions approaches were widely used for miRNA identification, and contributed greatly to the miRNA discovery [6]–[10]. However, in addition to the highly conserved miRNAs, some other miRNAs are only expressed in certain species, tissue or developmental stages and may accumulate at lower levels, which made it difficult to detect them with the traditional methods. The recent approach using next-generation high throughput sequencing technology provides a rapid and high throughput tool to explore the large inventory of sRNA populations and to identify low abundant miRNAs involved in specific processes. Since its use in the model species *Arabidopsis* [11]–[13], the next-generation high throughput sequencing technology has been successfully applied in many plant species [14]–[18], and the number of reported plant miRNAs is increasing rapidly. To date, a total of 2,109 plant miRNAs from 46 species have been identified and deposited in miRBase (miRBase Release 17.0, http://www.mirbase.org/).

To thoroughly understand the biological functions of miRNA, it is not only necessary to accurately identify the miRNAs, but also to predict the targets and explore their interactions. Usually, based on the perfect sequence complementarity between a miRNA and its target or the conservation of miRNA targets among different plant species, computational target prediction was widely employed in identifying plant miRNA targets [19], [20]. However, due to the existence of a higher mismatch in miRNA-target pairing, computational target prediction method is often questionable as concern to distinguishing the authenticity of predicted target genes [21]. Therefore, all the prediction targets should be confirmed by experimental approaches. So far, the modified 5′ RACE remains the most widely method for target confirmation and cleavage site mapping [22]. Duo to intensive labor work and high cost, this method is, however, only applicable to identify targets in small-scale, which greatly affects the efficiency of target validation. Fortunately, the recent advent of degradome sequencing opens up a new avenue for high-throughput validation of the splicing targets on a whole genome scale. The power of the method lies in permitting large-scale validation of small RNA targets, and, as a result, it has revolutionized the traditional target validation experimentation. Recently, it has been successfully applied to screen for miRNA targets in *Arabidopsis* [23], [24], rice [25], grapevine [18] and soybean [26].

Cucumber is one of the most important greenhouse species in the world. However, despite its economic and biological importance, and availability of the complete genome sequence, the number of miRNAs identified from cucumber plants is very limited as compared to other plant species [27]. Furthermore, there still has been no report of experimental validation of the related miRNA targets in cucumber, which is critical for understanding of the roles of miRNAs in cucumber. Therefore, further identification of specific expression of miRNAs in different tissues and developmental stages, as well as elucidation of their functions with experimental validation of the related miRNA targets will help us understand the regulatory mechanism of miRNAs in cucumber. The goal of this study is to identify tissue specific expression of miRNAs and their potential targets in cucumber. To achieve this goal, two independent small RNA libraries from cucumber leaves and roots were constructed and sequenced by the high-throughput Illumina Solexa system. A selected number of cucumber miRNAs were then validated by quantitative RT-PCR. Based on these newly identified cucumber miRNAs, we also predicted their potential miRNA targets by degradome sequencing for the first time in cucumber.

## Results

### The small RNA profile in cucumber

**Figure 1:**
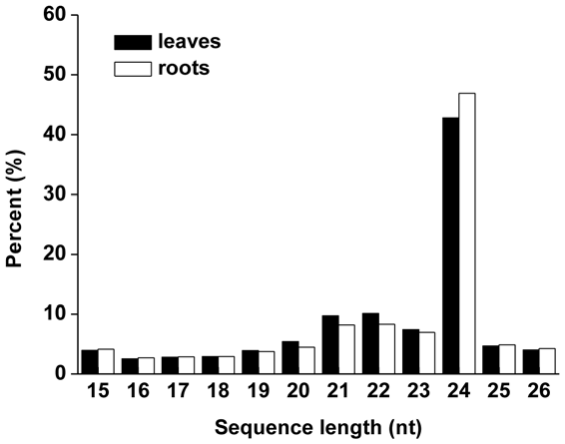
The size distribution of the small RNAs in leaves and roots libraries of cucumber.

To identify miRNAs in cucumber, two independent small RNA libraries from cucumber leaves and roots were sequenced with the high-throughput Illumina Solexa system, which generated a total of 6,055,873 and 7,574,396 raw reads, respectively. After filtering out the adapter sequences as well as sequences with low quality or low-copy (copy<3), 4,012,509 and 4,807,017 sequences were obtained with length 15-26 nt from leaves and roots, respectively (Table 1). After further removing mRNA, rRNAs, tRNAs, snRNAs, and snoRNAs, a total of 2,525,960 and 2,950,151 mappable small RNA sequences were obtained from leaves and roots, respectively (Table 1). In both libraries, the majority of the sRNAs were 20–24 nt in size, with 24 nt having the highest abundance (Fig. 1). These results were consistent with the typical small RNA distribution of angiosperms, such as rice [28], Medigcago [29], and cucumber [27].

**Table 1:** Statistics of small RNA sequences from the cucumber leaves and roots libraries.

Category	Leaves		Roots	
	Sequences	Unique sequences	Sequences	Unique sequences
Raw reads	6055873	1234981	7574396	1861481
Adaptor removed	131690	17128	125273	10973
Low quality reads removed	111585	27531	50734	42876
Sequences <15 nt, >26 nt filter	864176	240088	829401	182042
Copy<3 removed	935913	851993	1761971	1502957
mRNA, RFam, Repbase matches removed	1486549	32781	1856866	32083
Mappable sequences	2525960	65460	2950151	90550

Mappable sequences: The raw reads were passed through a series of the digital filters by Illumina's Genome Analyzer Pipeline software and ACGT101-miR program, and the resulting sequence were called “mappable sequences” [57].

### Identification of known miRNAs in cucumber

To identify miRNAs in cucumber, all the mappable sRNA sequences were first compared to the currently known plant miRNAs in miRBase v17 database. A total of the 60 known unique miRNAs with high sequence similarity to the known plant miRNAs were identified (Table S1). Most of these known miRNAs (68.3%) were 21 nt in length with the remainder being 20 nt or 22 nt long (Table S1). This is similar to observations of miRNAs from other plant species, indicating that cucumber miRNAs are mostly processed by DCL 1 [30].

Base on the sequence similarity, these identified miRNAs could be grouped into 29 miRNA families (Table 2 and Table S1). Most of the identified miRNA families such as miR156, miR159, miR167, miR394 and miR398 are highly conserved in a variety of plant species (Table S1). In addition, as expected, we also found several known but non-conserved miRNA (miR170, miR477, miR530, miR827, miR858, miR1515, miR2111, and miR2950) in our dataset that have previously been identified only from one or a few plant species. Based on the prediction of secondary structures, 13 potential precursors of known miRNA were identified in the cucumber genome, of which one miRNA^*^ sequences (csa-miR393-3P) had also been sequenced by deep sequencing (Table S1). The predicted hairpins have a minimal folding free energy (MFE) ranging from −37.3 kcal/mol to −82.8 kcal/mol and a minimal folding free energy index (MFEI) ranging from 0.85 to 1.32.

**Table 2:** Expression levels of cucumber miRNA families assessed using Solexa sequencing.

Family	Leave	Roots	Family	Leaves	Roots
csa-miR156/157	3304	1727	csa-miR393	43	25
csa-miR159	41206	223	csa-miR394	42	36
csa-miR160	360	1539	csa-miR396	2628	426
csa-miR162	277	298	csa-miR397	358	24840
csa-miR164	28	2127	csa-miR398	8046	5346
csa-miR166	442	3022	csa-miR399	14	25
csa-miR167	18070	16035	csa-miR408	5193	2030
csa-miR168	10687	8311	csa-miR477	3	88
csa-miR169	2434	1844	csa-miR530	555	176
csa-miR170	125	0	csa-miR827	14	45
csa-miR171	19	0	csa-miR858	11	0
csa-miR172	116	23	csa-miR1515	24	19
csa-miR319	6	5	csa-miR2111	38	160
csa-miR390	425	315	csa-miR2950	40	231

The expression level of cucumber miRNA families in each tissue was assessed by counting the number of all the reads mapping to each family, normalized by the total number of mappable sRNA in the respective libraries.

The expression profiles of miRNAs were analyzed and compared between two tissues based on the number of reads generated from the high-throughput sequencing. Interestingly, there was a significant difference of the relative abundance among the miRNA families. As showed in Table 2, miR167 and miR168 were abundant in both libraries, while several conserved miRNA (such as miR171, miR394, and miR399) as well as most of non-conserved miRNA, were found to have very low reads in both libraries or even to be undetectable in one library. However, we also found that some miRNAs were expressed preferentially in the leaves or roots. For example, the miR159 had higher abundance in leaves while miR160, miR164, miR166, and miR397 showed a higher expression level in roots, suggesting the potential functional divergence among the miRNA family.

### Identification of novel cucumber miRNAs

**Figure 2:**
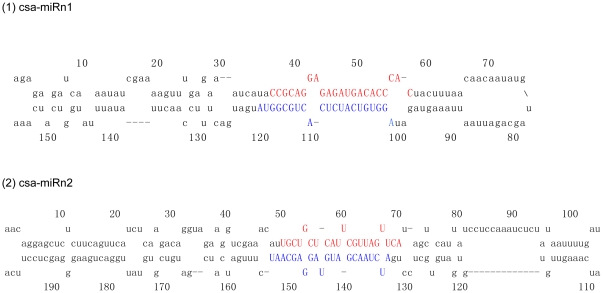
Predicted secondary structures of novel cucumber miRNAs. The mature miRNA and miRNA^*^ sequences are written with red and blue capital letters, respectively.

The ability of the miRNA flanking sequences to fold-back into a hairpin structure is an important criterion to differentiate candidate miRNA from other small RNAs. By mapping all unique sRNA sequences to the cucumber genome and predicting the hairpin structures for their flanking sequences, 332 miRNAs candidates were identified in this study. The MFE of these predicted pre-miRNAs ranged from −21.2 kcal/mol to −149.2 kcal/mol with an average of −52.9 kcal/mol, and MFEI ranged from 0.85 to 1.46 with an average of 1.08, which is apparently higher than other types of RNAs such as tRNAs (0.64), rRNAs (0.59) and mRNAs (0.62–0.66) [31]. These conditions meet the stability requirements of the secondary structure of miRNAs. Moreover, of these candidate miRNAs, two had miRNA^*^ sequences in the library (Table 3), and all these paired to their corresponding miRNAs with 2 nt 3′ overhangs (Fig. 2). Based on the recent annotation criteria of plant miRNAs [32], these two miRNAs were categorized as novel miRNAs in cucumber.

**Table 3:** Novel cucumber miRNAs identified by high-throughput sequencing.

miRNA name	Sequence (5′-3′)	LM	Precursor ID	LP	MFE(kcal/ml)	A+U%	MFEI	Frequency in leaves	Frequency in roots
csa-miRn1-5p	CCGCAGGAGAGATGACACCCAC	22	Scaffold001136	154	−58.2	64.9	1.08	0	25
csa-miRn1-3p	AGGTGTCATCTCACTGCGGTA	21	Scaffold001136	154	−58.2	64.9	1.08	0	5
csa-miRn2-5p	TGCTGCTCATTCGTTAGTTCA	21	Scaffold000215	198	−85.0	59.1	1.05	5	3
csa-miRn2-3p	ATCTAACGATGTAGGAGCAAT	21	Scaffold000215	198	−85,0	59.1	1.05	5	0

LM: length of the mature miRNA; LP: length of the miRNA precursor sequence; MFE: Minimal folding free energy; MFEI: Minimal folding free energy index

Frequency in leaves and roots: normalized sequencing frequencies in leaves and roots libraries, respectively.

### Confirmation of predicted miRNAs by qRT-PCR

**Figure 3:**
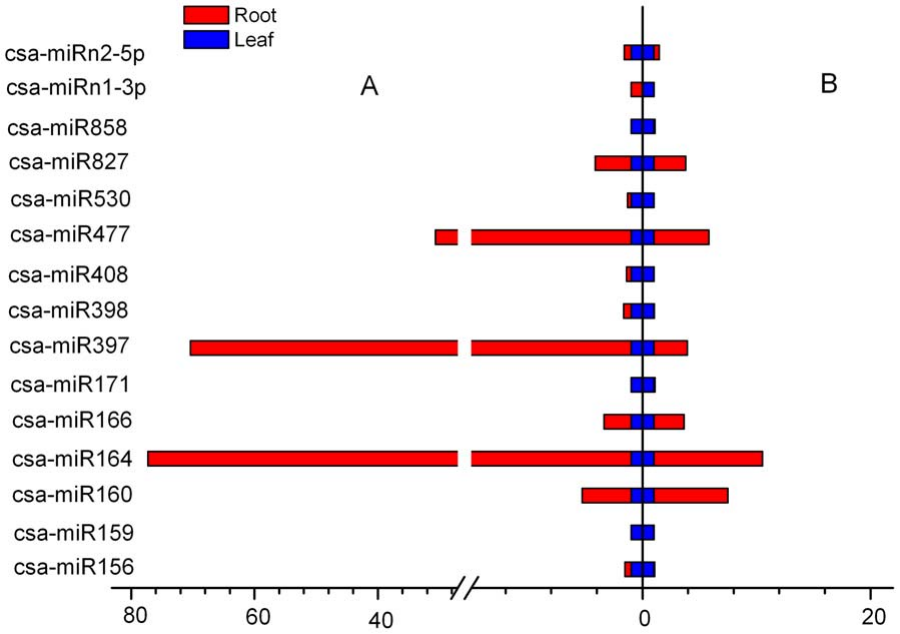
Expression analysis of miRNAs in cucumber leaves and roots by qRT-PCR. The amount of expression was normalized by the level of U6 in qRT-PCR. All reactions of qRT-PCR were repeated three times for each sample. Left indicates the miRNA relative expression generated from the high-throughput sequencing; Right indicates the miRNA relative expression tested by qRT-PCR.

To verify the existence and expression patterns of the predicted miRNAs, two novel miRNAs, as well as 13 representative known miRNAs displaying differential expression pattern in leaves and roots from the high-throughput sequencing were selected for qRT-PCR analysis. Although some non-conserved and new miRNAs were identified in low read number or undetectable by Solexa sequencing, all of them were detected by qRT-PCR. Overall, except for a few low-abundantly expressed miRNAs, most of the qRT-PCR results of the high abundance miRNAs were quite consistent with the results from sequencing data (Fig. 3). In particular, miR156 miR159, miR171, miR398, miR408, miR530 and miR858 were more abundant in leaves, whereas the abundances of miR160, miR164, miR166, miR397, miR477 and miR827 were higher in roots. Interestingly, miRn1-3p and miRn2-5p also showed a tissue-specific pattern, with a higher abundance in leaves (Fig. 3).

### Target identification for cucumber miRNAs by degradome analysis

**Figure 4:**
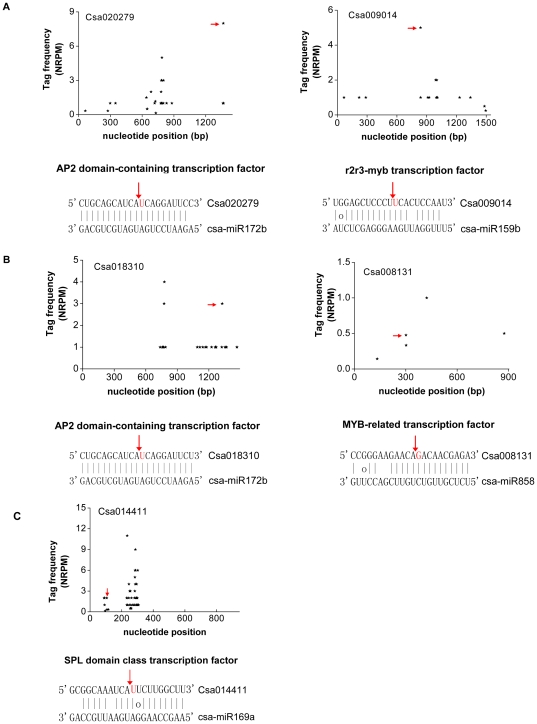
Target plots (t-plots) of miRNA targets in different categories confirmed by degradome sequencing. (A) T-plot (top) and miRNA: mRNA alignments (bottom) for two category I targets, Csa020279 and Csa009014 transcripts. The arrow indicates signatures consistent with miRNA-directed cleavage. The solid lines and dot in miRNA: mRNA alignments indicate matched RNA base pairs and GU mismatch, respectively, and the red letter indicates the cleavage site. (B) As in (A) for Csa18310 and Csa008131, a category II target for csa-miR172 and csa-miR858. (C) As in (A) for Csa014411, a category III target for csa-miR169.

To understand the potential biological function of these identified miRNAs, a recently developed degradome sequencing approach [23], [24] was applied to identify the targets of cucumber miRNAs. A total of 18650451 short sequencing reads representing the 3′cleavage fragment were generated. After initial processing and analyzing by CleaveLand 2.0 [33], a total of 21 genes targeted by 11 known miRNAs families were identified, of which 17 target genes were cleaved by 10 conserved miRNA families (including miR156/157, miR159, miR164, miR167, miR169, miR172, miR319, miR393 and miR398) and only 4 target genes were cleaved by miR858, a known but non-conserved miRNA family. Interestingly, we also found a miRNA pairs (miR156 and miR157) targeting the same gene Csa018095 (Fig. S1 and Table 4). Unfortunately, we could not detect the cleavage signature for most of known miRNA families and newly identified novel miRNA families in this degradome library. Based on the signature abundance at the target sites, these cleaved targets were classified into three categories (categories I, II and III) (Table 4) as previously described [23], [33]. Among these identified targets, twelve belonged to category I, eight were in category II, and only one was in category III. These results indicated that most of these targets were efficiently cleaved by miRNA. All the ‘target plots’ (t-plots) of identified targets were showed in Fig. 4 and Fig. S1.

**Table 4:** Cucumber miRNA targets identified by degradome sequencing.

miRNA family	Target gene family	Target gene accession	Cleavage site	Abundance	Category	Conserved in *Arabidopsis* *
csa-miR156	DNA primase large subunit	Csa008446	2338	0.75	II	
	squamosa promoter-binding protein	Csa018095	787	1	II	Y
csa-miR157	squamosa promoter-binding protein	Csa018095	786	1	II	
csa-miR159	r2r3-myb transcription factor	Csa009014	838	5	I	Y
csa-miR164	Single-stranded nucleic acid binding R3H	Csa013305	253	1	I	
csa-miR167	putative chloroplast chlorophyll a/b-binding protein	CO995238	26	0.54	I	
csa-miR169	SPL domain class transcription factor	Csa014411	106	2	III	
csa-miR172	AP2 domain-containing transcription factor	Csa010225	1423	3	I	Y
	AP2 domain-containing transcription factor	Csa012456	1234	7	II	Y
	AP2 domain-containing transcription factor	Csa018310	1327	3	II	Y
	AP2 domain-containing transcription factor	Csa020279	1366	8	I	Y
	Predicted membrane protein (ISS)	Csa007404	540	1	II	
csa-miR319	ATP binding protein, putative	Csa017286	455	4	II	
	MdTCP2B	CU7286	455	18	I	Y
csa-miR393	auxin signaling F-box 2	Csa015043	1513	6.20	I	Y
csa-miR398	Blue copper protein precursor	CU27969	55	2	I	
	Cu/Zn superoxide dismutase 2	DQ178941	176	1.75	I	Y
csa-miR858	MYB-related transcription factor	Csa008131	301	0.48	II	Y
	MYB transcription factor MYB161	Csa009345	304	0.67	I	Y
	ubiquitin ligase protein cop1	Csa012814	2071	15	I	
	R2R3 transcription factor MYB108-like protein	CU60428	48	0.5	I	Y

* According to Addo-Quaye et al. [23].

Based on the BLASTX analysis, 57.1% of the identified miRNA targets were generally homologous to conserved target genes that have already been found in other plants species. Most of these conserved target genes were transcription factors, including growth regulating factors [squamosa promoter binding (SBP) transcription factors, MYB transcription factors, AP2-like transcription factor, TCP transcription factors], and auxin response factors (auxin signaling F-box 2). These factors had been found to be involved in plant growth and/or responses to environmental changes in previous reports [34], [35]. Among them, mRNA for Cu/Zn superoxide dismutase which was confirmed as miR398 targets in *Arabidopsis* [23], [24], rice [25], and soybean [26] was also found to be cleaved by miR398 in this study. Interestingly, in addition to the well-documented conserved targets, we also identified some nonconserved targets regulated by known miRNAs. For instance, miR167 was found potentially to target a gene encoding chlorophyll a/b-binding protein (Table 4). As for miR398, besides targeting Cu/Zn superoxide dismutase, it also targeted a gene encoding blue copper protein precursor, which act as mobile electron carriers in a variety of biological systems. These results strongly suggest that the identified cucumber miRNAs regulate a wide range of genes not only in development but also in other physiological processes.

#### GO function analysis of targets

To better understand miRNA functions, we subjected the identified target genes to Gene Ontology (GO) analysis, a promising method for uncovering the miRNA-gene regulatory network on the basis of biological process and molecular function [36]. The result of GO analysis demonstrated that the 21 predicted targets could be classified into 36 biological processes, and 11 miRNA families were found to take part in a broad range of physiological processes, including transcription regulation, cell differentiation, organismal development, vegetative to reproductive phase transition, photosynthesis, defense against stresses, hormone stimulus and light signaling pathways (Table 5). Many miRNA families were involved in the same biological process. For example, miR156, miR157, miR159, miR169, miR172 and miR858 participated in transcription regulation while miR159, miR172, miR393 and miR858 participated in multicellular organismal development.

**Table 5:** GO analyses show that miRNAs potentially target tissue forming-related biological processes.

miRNAs	GO Biological Process	gene	Total numberof target
miR156,157,159,169,172,858	transcription	Csa008446,Csa018095,Csa009014,Csa014411,Csa010225,Csa012456,Csa018310, Csa020279,Csa008131,Csa009345,CU60428	11
miR159	regulation of gene expression	Csa009014	1
miR159,172,393,858	multicellular organismal development	Csa009014,Csa010225,Csa012456,Csa018310,Csa020279,Csa007404,Csa015043,Csa009345	8
miR159,172	flower development	Csa009014,Csa010225,Csa012456,Csa018310,Csa020279	5
miR172	specification of floral organ identity	Csa010225,Csa012456,Csa018310,Csa020279	4
miR172	meristem maintenance	Csa010225,Csa012456,Csa018310,Csa020279	4
miR393	lateral root formation	Csa015043	1
miR172	vegetative to reproductive phase transition	Csa010225,Csa012456,Csa018310,Csa020279	4
miR159,172	cell differentiation	Csa009014, Csa010225,Csa012456,Csa018310,Csa020279	5
miR858	red or far red light signaling pathway	Csa012814,CU60428	2
miR858	negative regulation of photomorphogenesis	Csa012814	1
miR858	photomorphogenesis	Csa012814	1
miR319 ,858	response to stress	Csa017286	1
miR393	defense response	Csa015043	1
miR398	response to oxidative stress	CU27969	1
miR398	response to absence of light	CU27969	1
miR159	response to salt stress	Csa009014	1
miR159	response to wounding	Csa009014	1
miR393	cellular response to phosphate starvation	Csa015043	1
miR172,393	ethylene mediated signaling pathway	Csa010225,Csa012456,Csa018310,Csa020279,Csa015043	5
miR858	response to ethylene stimulus	CU60428	1
miR393	response to auxin stimulus	Csa015043	1
miR393	auxin mediated signaling pathway	Csa015043	1
miR159,858	response to abscisic acid stimulus	Csa009014,Csa008131,Csa009345	3
miR858	response to gibberellin stimulus	Csa009345,CU60428	2
miR159,858	response to salicylic acid stimulus	Csa009014,Csa009345,CU60428	3
miR858	response to jasmonic acid stimulus	Csa009345,CU60428	2
miR319,858	protein amino acid phosphorylation	Csa017286,Csa012814	2
miR167	photosynthesis, light harvesting	CO995238	1
miR167	photosynthesis	CO995238	1
miR398	aluminum ion transport	CU27969	1
miR398	electron transport chain	CU27969	1
miR393,858	modification-dependent protein catabolic process	Csa015043,Csa012814	2
miR398	oxidation reduction	DQ178941	1
miR159	cinnamic acid biosynthetic process	Csa009014	1
miR159	flavonoid biosynthetic process	Csa009014	1

## Discussion

Identification of miRNAs and their targets is the basis for understanding the physiological functions of miRNAs. Many plant miRNAs have been deposited to miRBase and their physiological functions have also been studied. The research on cucumber miRNAs, however, is just in its infancy. The recent completion of the sequence of the cucumber genome provides a powerful resource for identification of cucumber miRNAs [37]. Based on the sequence of the cucumber genome, 49 mature miRNA belonging to 25 known miRNA families as well as 7 new miRNA families were detected by deep sequencing in a cucumber library generated from phloem exudate and leaves of cucumber infected with *Hop stunt viroid* [27]. However, experimental validation of the miRNA targets was not carried out, which greatly hindered the research of the miRNAs regulation mechanism in cucumber. In this study, we expanded cucumber miRNA data set by identifying 60 known miRNAs as well as 2 new miRNAs with their miRNA^*^ star strands, of which 37 known miRNAs and all the new miRNAs were firstly revealed in cucumber. Moreover, we for the first time revealed 21 potential targets of these miRNAs by the recently developed degradome sequencing approach. This will offer new opportunities for the revelation of the miRNA-mediated transcriptional regulatory networks in cucumber.

A wide range of characteristics were featured in these newly identified known miRNAs in cucumber. As reported by Martínez et al. [27], most of the identified known miRNA families are highly evolutionarily conserved in a variety of plant species (Table S1). For example, miR156/157, miR319, miR165/166, miR169, miRNA 394 and miR172 have been found to have orthologs in 45, 51, 40, 41, 40 and 24 kinds of plant species, respectively [31], [38], suggesting that these miRNAs play important and conserved roles in plant kingdom. As for known but non-conserved miRNAs, in addition to miR170, miR827, miR858 and miR2950 which have been reported in cucumber [27], four other miRNA families (miR477, miR530, miR1515 and miR2111) were for the first time detected by this study. Of these four miRNA families, miR1515 has been only identified in *Citrus sinensis* and *Glycine max* so far. It seems likely that these miRNAs relatively recently evolved [22], and play important roles in more species-specific characteristics in plant growth and development [31]. Therefore, although presenting at low level, these non-conserved miRNAs might play species-specific functions in cucumber.

Analyzing the spatial and temporal expression patterns of miRNAs would provide useful information about their physiological functions [30]. In plants, increasing evidence showed that many miRNAs have differential accumulation in specific developmental stages and tissues [39]. For example, miR159, which is considered to have crucial function in leaf development, accumulated mainly in the leaf as compared to other tissues in potato [40]. On the other hand, several miRNA families such as miR164 and miR390, have an essential role in plant root development including root cap formation, lateral root development, or adventitious rooting through their ARF (auxin response factor) targets-mediated downstream pathways [41], [42]. MiR164 showed a significant higher expression in roots than in leaves in several plant species [41], [43]. In addition, the recent studies also showed that miR397 was more abundant in opium poppy leaves, and miR171 was higher in opium poppy roots, while miR156 and miR408 were more abundant in barley leaves, and miR166 was higher in barley roots [44], [45]. Based on the sequencing reads and identification by qRT-PCR, many miRNAs also showed differential expression in different tissues in our study. Consistent with previous reports, miR164 and miR166 were highly abundant in cucumber roots, while miR156, miR159, and miR408 were highly abundant in cucumber leaves. On the other hand, we found miR397 and miR171 was highly abundant in roots and leaves, respectively, which were quite different from the patterns found in opium poppy [44]. This suggests that in addition to some common mechanism shared by different plant species, there are species-specific miRNA regulatory mechanisms in cucumber miRNA.

In addition to tissue-specific miRNA, many miRNAs have been demonstrated to be responsive to growth stages and growth conditions. A series of recent reports found that environmental stress-related miRNA were mostly suppressed in plants grown under normal conditions. For example, miR395 were usually undetectable in normal plants but induced strongly under sulphate starvation [21]. MiR399 was specifically induced under low phosphate stress [46], while miR393 levels were increased by a variety of stresses [47]. Consistent with these reports, miR393, and miR399 showed a significantly lower level of expression in this study. However, whether these miRNAs identified in this study would express in other tissues, or whether they are responsive to biotic or abiotic stress, remains to be investigated.

Based on deep sequencing and the hairpin structure prediction, we were able to identify two novel miRNAs with their miRNA^*^ star strands, an essential requirement for novel miRNA prediction [32]. Because these miRNAs were not similar to any known miRNAs, they might be specific to cucumber and play more specific roles. As previously observed in other plants, these novel miRNAs were expressed at low levels and difficult to detect [12], [13]. All of these novel cucumber miRNAs were validated in this study and showed their preferential expression in leaves as revealed by qRT-PCR (Fig. 3) which might provide important clues about their physiological functions.

Identification of target gene with accuracy is essential to reveal the regulatory networks of miRNA. Previous work on the identification is limited to the bioinformational prediction [27], and is therefore not adequate. In this study, we identified 21 potential targets for 11 known miRNA families in cucumber by degradome sequencing, an efficient strategy to identify target genes of miRNAs [23], [24] (Table 4). Among these targets, 57.1% belonged to category I, suggesting that miRNA was the key regulator of these genes [26]. Interestingly, we also found that the same member of the SBP family (Csa018095) was cleaved by a pairs of miRNAs (miR156 and miR157; Fig. S1 and Table 4), suggesting that there was a combinatorial genes regulation pathway involving a pair of miRNAs in cucumber [18].

Consistent with other plant species, most conserved miRNA families were shown to be more likely to target transcription factors involved in regulating plant growth and development. In our study, mRNAs for squamosa promoter binding (SBP) transcription factors, MYB transcription factors, AP2-like transcription factor, TCP transcription factors, and auxin signaling F-box 2 were cleaved by miR156/157, miR159/858, miR172 miR319 and miR393 families, respectively. All of these transcription factors played an important role in plant growth and development. For example, by negatively regulating SBP transcription factors and AP2-like transcription factors, respectively, miR156 and miR172 regulated juvenile-to-adult vegetative phase transition in plant [48]. Overexpression of miR319a resulted in the degradation of *TCPs* and delayed the leaf senescence [49]. In addition to targeting transcription factors, some miRNAs in cucumber were also shown to be involved in stress response and metabolic processes, including miR398, which targeted Cu/Zn-SODs, a well-known protein functioning in mitigating oxidative stress during biotic and abiotic stress [50]. The similarity of conserved targets to *Arabidopsis*, rice and grave, suggests that these miRNA-mediated plant regulatory mechanisms might be conserved through plant kingdom. Interestingly, several non-conserved targets including chlorophyll a/b-binding protein and blue copper protein precursor were also validated as genuine targets of miR167 and miR398, respectively. Chlorophyll a/b-binding protein is well known for its role in plant photosynthesis, and blue copper protein has been reported to function as electron carriers in a variety of biological systems. In order to more thoroughly understand the function of miRNA, we further found that 21 of these target genes belonging to 11 miRNA families were involved in 36 physiological processes through GO analysis. These physiological processes include not only transcription regulation, organismal development, vegetative to reproductive phase transition, photosynthesis, defense against stresses, hormone stimulus discussed above, but also cell differentiation, light signaling pathway, cinnamic acid biosynthetic process and flavonoid biosynthetic process. Interestingly, as previously reported [35], there were also several miRNA regulatory groups in cucumber that are involved in the same physiological processes including transcription, cell differentiation, multicellular organismal development, flower development and ethylene mediated signaling pathway. It suggests that these miRNA regulatory groups participate in the same physiological processes by interacting with each other. For example, in the developmental processes, miR159 and miR172 might co-participate in cell differentiation and flower development. In response to stress, miR159 and miR858 might co-participate in the response to abscisic acid and salicylic acid stimulus, while ethylene-mediated signaling pathway might be regulated by miR172 and miR393.

It is also worth to note that not all the cucumber miRNAs including conserved and novel miRNAs were found to have their detectable sliced targets in this study. As discussed above, miRNAs are differentially expressed in tissue-specific and stage-specific manners, and the degradome abundances of some targets may not be sufficient to be detectable in leaf tissues. On the other hand, besides transcript cleavage, miRNAs have also been shown to regulate their targets by translational repression [51], and targets regulated by such a mode would be undetectable by degradome sequencing. In addition, the lack of completely mRNA data may also limit the comprehensive identification of targets in some extent. Therefore, further construction of degradome libraries from different tissues, organs and different developmental stages would provide more insight into the interaction between miRNAs and their corresponding targets.

In summary, we have not only identified 64 cucumber miRNAs, which belongs to 29 known and two novel miRNA families, but also discovered that some of the miRNAs are differentially expressed in a tissue-dependent manner. For the first time, we detected 21 sliced targets, which reveal interaction between miRNA and target in cucumber by using the recently developed degradome sequencing approach. This report will offer a foundation for future studies of the miRNA-mediated regulatory networks in cucumber.

## Methods

### Small RNA library construction and sequencing

The leaves and roots of cucumber (*Cucumis sativus* L. cv. Jinchun No. 2) were collected at the two-leaf stage,and frozen in liquid nitrogen immediately, and then stored at −80°C until RNA isolation. For small RNA library construction,all the three different samples were pooled and used to extract total RNA with *mir*Vana miRNA Isolation Kit (Ambion, Austin, TX, USA) according to the manufacturer's instructions. About 10 µg of small RNA were used for sequencing by the Genome Analyzer GA-I (Illumina, San Diego, USA) following the manufacturer's protocols. In brief, the sRNA fractions with the length of 10–40 nt were isolated by 15% denaturing polyacrylamide gel electrophoresis. After ligating with 5′ and 3′ adaptors, the obtained short RNAs were reversely transcribed to cDNA according to the Illumina protocol. The resulting small RNA libraries were then sequenced by the Genome Analyzer GA-I (Illumina, San Diego, USA).

### Identification of known and novel miRNAs

The raw sequences were firstly processed by Illumina's Genome Analyzer Pipeline software to filter out the adapter sequences, low quality as well as low-copy sequences, and then the extracted small RNA sequences with 15–26 nt in length were subjected to mRNA, RFam, Repbase filter. Finally, the remaining unique sequences were compared to the miRNA database, miRBase 17.0 (http://www.mirbase.org/) by BLASTn search to identify the conserved miRNAs in cucumber. A maximum of three mismatches were allowed between identified cucumber miRNAs and currently known plant miRNAs [52].

To identify potential miRNA precursor sequences, all identified cucumber mature miRNA sequences were further BLASTed against the cucumber draft genome sequences which downloaded from cucumber genome database (http://cucumber.genomics.org.cn/) and predicated for the hairpin RNA structures for their flanking sequences by UNAfold software (http://rna.tbi.univie.ac.at/cgi-bin/RNAfold.cgi). Non-coding sequences, which met previously described criteria were then considered to be a potential miRNA precursor. Specifically, (1) the identified miRNAs were located in the arms of stem-loop structure; (2) no large loop or break were exist in the identified miRNA sequence; (3) a maximum of six mismatches were allowed between the identified miRNAs and the opposite miRNA sequence (miRNA*); (4) the potential miRNA precursor must have higher negative minimal folding energy (MFE) and minimal free folding energy indexes (MFEI) to distinguish from other small RNAs [31], [52].

To identify potential novel miRNAs in cucumber, the rest unmapped small RNA sequences were also BLASTed against the genome and folded into a secondary structure as above. Only the non-coding sequences which could form a perfect stem-loop structure and meet the criteria for miRNAs prediction [32] were then considered to be a potential novel miRNA candidate.

### Verification of cucumber miRNAs by quantitative real-time PCR (qRT-PCR)

To validate the presence and expression of the identified miRNAs, 13 known miRNAs and two cucumber novel miRNAs were selected for qRT-PCR. Total RNA was extracted from leaves and roots using Trizol (Invitrogen) according to the manufacturer's instructions, and then treated with RNase-free DNase I (TaKaRa, Dalian, China) to remove the genomic DNA. The specific forward primers of 15 selected miRNAs were designed according to the sequence of miRNA itself, which were available in Table S2. The reverse transcription reaction was performed with the One Step PrimeScript®miRNA cDNA Synthesis Kit (TaKaRa, Dalian, China) according to the manufacturer's protocol [53]

The qRT-PCR was performed with SYBR Premix Ex Taq II (TaKaRa, Dalian, China) on the iCycler iQ real-time PCR detection system (Bio-Rad). All reactions were performed in triplicate for each sample and U6 snRNA was used as an internal reference. The relative expression level of miRNA was calculated according to the method of Livak and Schmittgen [54].

### Degradome library construction and target identification

To predict the potential target mRNAs, a degradome library was constructed from cucumber leaves as previously described by German et al. [24], [55]. Briefly, polyA-enriched RNA molecules were isolated and ligated to an RNA oligonucleotide adaptor containing a 3′ MmeI recognition site,the ligated products were used to generate first-strand cDNA by reverse transcription (RT). Then a short PCR was used to amplify the cDNA to obtain sufficient quantities of DNA products. After purification and digestion with MmeI, the PCR product was ligated to a double-stranded DNA adaptor, and then gel purified again for PCR amplification. The final cDNA library was purified and sequenced on Illumina GAIIx following vendor's instruction.

Raw sequencing reads were obtained using Illumina's Pipeline v1.5 software to remove adaptor sequences and low quality sequencing reads. The extracted sequencing reads with the length of 20 and 21 nt were then used to identify potentially cleaved targets by the CleaveLand pipeline as previously described [23], [33]. The degradome reads were mapped to the cucumber sequences of mRNA and EST downloaded from Cucurbit Genomics Datebase (http://www.icugi.org/) and NCBI (http://www.ncbi.nlm.nih.gov/). Only the perfect matching alignment(s) for the given read would be kept and extend to 35–36 nt by adding 15 nt of upstream of the sequence. All resulting reads (t-signature) were reverse-complemented and aligned to the miRNA identified in our study. No more than five mismatches of the alignments were allowed. Alignments where the 5′ the degradome sequence position coincident with the tenth nucleotide of miRNA were retained and scored by previously described method [56]. The target was selected and categorized as I, II, or III as previous study [23]. In addition, to easily analyze the miRNA targets and RNA degradation patterns, t-plots were built according to the distribution of signatures (and abundances) along these transcripts. All the identified targets were subjected to BlastX analysis to search for similarity, and then to GO analysis to uncover the miRNA-gene regulatory network on the basis of biological process and molecular function as previously described by Xie et al. [35] .

## Supporting Information

Figure S1 **Target plots (t-plots) of miRNA targets confirmed by degradome sequencing.** Signature abundance is plotted as the length of the transcript. The miRNA-directed cleavage signature is shown as the red arrow. The red letter in miRNA:mRNA alignments indicates the cleavage site detected in the degradome.
(TIF)

Table S1 **Known miRNAs identified in cucumber and their sequence similarity to known miRNAs from other plant species.**
(DOC)

Table S2 **Primer sequences used for qRT-PCR.**
(DOC)

## References

[1] Llave C, Kasschau KD, Rector MA, Carrington JC (2002). Endogenous and silencing-associated small RNAs in plants.. Plant Cell.

[2] Bartel DP (2004). MicroRNAs: genomics, biogenesis, mechanism, and function.. Cell.

[3] Sunkar R, Chinnusamy V, Zhu JH, Zhu JK (2007). Small RNAs as big players in plant abiotic stress responses and nutrient deprivation.. Trends Plant Sci.

[4] Shukla LI, Chinnusamy V, Sunkar R (2008). The role of microRNAs and other endogenous small RNAs in plant stress responses.. Biochim Biophys Acta-Gene Regul Mech.

[5] Chuck G, Candela H, Hake S (2009). Big impacts by small RNAs in plant development.. Curr Opin Plant Biol.

[6] Bonnet E, Wuyts J, Rouze P, Van de Peer Y (2004). Detection of 91 potential conserved plant microRNAs in *Arabidopsis thaliana* and *Oryza sativa* identifies important target genes.. Proc Natl Acad Sci USA.

[7] Adai A, Johnson C, Mlotshwa S, Archer-evans S, Manocha V (2005). Computational prediction of miRNAs in *Arabidopsis thaliana*.. Genome Res.

[8] Williams L, Carles CC, Osmont KS, Fletcher JC (2005). A database analysis method identifies an endogenous trans-acting short-interfering RNA that targets the *Arabidopsis ARF_2_*, *ARF_3_*, and *ARF_4_* genes.. Proc Natl Acad Sci USA.

[9] Zhang BH, Pan XP, Wang QL, Cobb GP, Aanderson TA (2005). Identification and characterization of new plant microRNAs using EST analysis.. Cell Res.

[10] Pilcher RLR, Moxon S, Pakseresht N, Moulton V, Manning K (2007). Identification of novel small RNAs in tomato (*Solanum lycopersicum*).. Planta.

[11] Lu C, Tej SS, Luo SJ, Haudenschild CD, Meyers BC (2005). Elucidation of the small RNA component of the transcriptome.. Science.

[12] Rajagopalan R, Vaucheret H, Trejo J, Bartel DP (2006). A diverse and evolutionarily fluid set of microRNAs in *Arabidopsis thaliana*.. Genes Dev.

[13] Fahlgren N, Howell MD, Kasschau KD, Chapman EJ, Sullivan CM (2007). High-throughput sequencing of *Arabidopsis* microRNAs: Evidence for frequent birth and death of MIRNA Genes.. PLoS ONE.

[14] Subramanian S, Fu Y, Sunkar R, Barbazuk WB, Zhu JK (2008). Novel and nodulation-regulated microRNAs in soybean roots.. BMC Genomics.

[15] Sunkar R, Zhou XF, Zheng Y, Zhang WX, Zhu JK (2008). Identification of novel and candidate miRNAs in rice by high throughput sequencing.. BMC Plant Biol.

[16] Arenas-Huertero C, Perez B, Rabanal F, Blanco-Melo D, De la Rosa C (2009). Conserved and novel miRNAs in the legume *Phaseolus vulgaris* in response to stress.. Plant Mol Biol.

[17] Lelandais-Briere C, Naya L, Sallet E, Calenge F, Frugier F (2009). Genome-wide *Medicago truncatula* small RNA analysis revealed novel microRNAs and isoforms differentially regulated in roots and nodules.. Plant cell.

[18] Pantaleo V, Szittya G, Moxon S, Miozzi L, Moulton V (2010). Identification of grapevine microRNAs and their targets using high-throughput sequencing and degradome analysis.. Plant J.

[19] Rhoades MW, Reinhart BJ, Lim LP, Burge CB, Bartel B (2002). Prediction of plant microRNA targets.. Cell.

[20] Chaudhuri K, Chatterjee R (2007). MicroRNA detection and target prediction: Integration of computational and experimental approaches.. DNA Cell Biol.

[21] Jones-Rhoades MW, Bartel DP (2004). Computational identification of plant microRNAs and their targets, including a stress-induced miRNA.. Mol Cell.

[22] Jones-Rhoades MW, Bartel DP, Bartel B (2006). MicroRNAs and their regulatory roles in plants.. Annu Rev Plant Biol.

[23] Addo-Quaye C, Eshoo TW, Bartel DP, Axtell MJ (2008). Endogenous siRNA and miRNA targets identified by sequencing of the *Arabidopsis* degradome.. Curr Biol.

[24] German MA, Pillay M, Jeong DH, Hetawal A, Luo SJ (2008). Global identification of microRNA-target RNA pairs by parallel analysis of RNA ends.. Nat Biotechnol.

[25] Li YF, Zheng Y, Addo-Quaye C, Zhang L, Saini A (2010). Transcriptome-wide identification of microRNA targets in rice.. Plant J.

[26] Song QX, Liu YF, Hu XY, Zhang WK, Ma BA (2011). Identification of miRNAs and their target genes in developing soybean seeds by deep sequencing.. BMC Plant Biol.

[27] Martínez G, Forment J, Llave C, Pallás V, Gómez G (2011). High-throughput sequencing, characterization and detection of new and conserved cucumber miRNAs.. PLoS ONE.

[28] Morin RD, Aksay G, Dolgosheina E, Ebhardt HA, Magrini V (2008). Comparative analysis of the small RNA transcriptomes of *Pinus contorta* and *Oryza sativa*.. Genome Res.

[29] Szittya G, Moxon S, Santos DM, Jing R, Fevereiro MPS (2008). High-throughput sequencing of *Medicago truncatula* short RNAs identifies eight new miRNA families.. BMC Genomics.

[30] Wang L, Wang MB, Tu JX, Helliwell CA, Waterhouse PM (2007). Cloning and characterization of microRNAs from *Brassica napus*.. FEBS Lett.

[31] Zhang BH, Pan XP, Cox SB, Cobb GP, Anderson TA (2006). Evidence that miRNAs are different from other RNAs.. Cell Mol Life Sci.

[32] Meyers BC, Axtell MJ, Bartel B, Bartel DP, Baulcombe D (2008). Criteria for annotation of plant microRNAs.. Plant Cell.

[33] Addo-Quaye C, Miller W, Axtell MJ (2009). CleaveLand: a pipeline for using degradome data to find cleaved small RNA targets.. Bioinformatics.

[34] Unver T, Budak H (2009). Conserved microRNAs and their targets in model grass species *Brachypodium distachyon*.. Planta.

[35] Xie FL, Frazier TP, Zhang BH (2011). Identification, characterization and expression analysis of MicroRNAs and their targets in the potato (*Solanum tuberosum*).. Gene.

[36] Ashburner M, Bergman CM (2005). Drosophila melanogaster: a case study of a model genomic sequence and its consequences.. Genome Res.

[37] Huang SW, Li RG, Zhang ZH, Li L, Gu XF (2009). The genome of the cucumber, *Cucumis sativus* L.. Nature Genet.

[38] Sunkar R, Jagadeeswaran G (2008). In silico identification of conserved microRNAs in large number of diverse plant species.. BMC Plant Biol.

[39] Sunkar R, Girke T, Jain PK, Zhu JK (2005). Cloning and characterization of microRNAs from rice.. Plant Cell.

[40] Yang WZ, Liu X, Zhang JG, Feng JL, Li C (2010). Prediction and validation of conservative microRNAs of *Solanum tuberosum* L.. Mol Biol Rep.

[41] Guo HS, Xie Q, Fei JF, Chua NH (2005). MicroRNA directs mRNA cleavage of the transcription factor NAC1 to downregulate auxin signals for *Arabidopsis* lateral root development.. Plant Cell.

[42] Yoon EK, Yang JH, Lim J, Kim SH, Kim SK (2010). Auxin regulation of the microRNA390-dependent transacting small interfering RNA pathway in *Arabidopsis* lateral root development.. Nucleic Acids Res.

[43] Mallory AC, Dugas DV, Bartel DP, Bartel B (2004). MicroRNA regulation of NAC-domain targets is required for proper formation and separation of adjacent embryonic, vegetative, and floral organs.. Curr Biol.

[44] Unver T, Parmaksız I, Dündar E (2010). Identification of conserved micro-RNAs and their target transcripts in opium poppy (*Papaver somniferum* L.).. Plant Cell Rep.

[45] Kantar M, Unver T, Budak H (2010). Regulation of barley miRNAs upon dehydration stress correlated with target gene expression.. Funct Integr Genomics.

[46] Fujii H, Chiou TJ, Lin SI, Aung K, Zhu JK (2005). A miRNA involved in phosphate-starvation response in *Arabidopsis*.. Curr Biol.

[47] Sunkar R, Zhu JK (2004). Novel and stress-regulated microRNAs and other small RNAs from *Arabidopsis*.. Plant Cell.

[48] Jung JH, Seo YH, Seo PJ, Reyes JL, Yun J (2007). The GIGANTEA-regulated microRNA172 mediates photoperiodic flowering independent of CONSTANS in *Arabidopsis*.. Plant Cell.

[49] Schommer C, Palatnik JF, Aggarwal P, Chetelat A, Cubas P (2008). Control of jasmonate biosynthesis and senescence by miR319 targets.. PLoS Biol.

[50] Jagadeeswaran G, Saini A, Sunkar R (2009). Biotic and abiotic stress down-regulate miR398 expression in *Arabidopsis*.. Planta.

[51] Brodersen P, Sakvarelidze-Achard L, Bruun-Rasmussen M, Dunoyer P, Yamamoto YY (2008). Widespread translational inhibition by plant miRNAs and siRNAs.. Science.

[52] Yin ZJ, Li CH, Han ML, Shen FF (2008). Identification of conserved microRNAs and their target genes in tomato (*Lycopersicon esculentum*).. Gene.

[53] Wang TZ, Chen L, Zhao MG, Tian QY, Zhang WH (2011). Identification of drought-responsive microRNAs in *Medicago truncatula* by genome-wide highthroughput sequencing.. BMC Genomics.

[54] Livak KJ, Schmittgen TD (2001). Analysis of relative gene expression data using real-time quantitative PCR and the 2^−ΔΔCT^ method.. Methods.

[55] German MA, Luo SJ, Schroth G, Meyers BC, Green PJ (2009). Construction of Parallel Analysis of RNA Ends (PARE) libraries for the study of cleaved miRNA targets and the RNA degradome.. Nat Protoc.

[56] Allen E, Xie ZX, Gustafson AM, Carrington JC (2005). MicroRNA-directed phasing during trans-acting siRNA biogenesis in plants.. Cell.

[57] Li MZ, Xia YL, Gu YR, Zhang K, Lang QL (2010). MicroRNAome of porcine pre- and postnatal development.. PLoS ONE.

