# Supplementary figures and images for: A Combined Approach of High-Throughput Sequencing and Degradome Analysis Reveals Tissue Specific Expression of MicroRNAs and Their Targets in Cucumber
Source: PLoS One. 2012 Mar 30;7(3):e33040. doi: 10.1371/journal.pone.0033040 (PMC3316546; doi:10.1371/journal.pone.0033040)

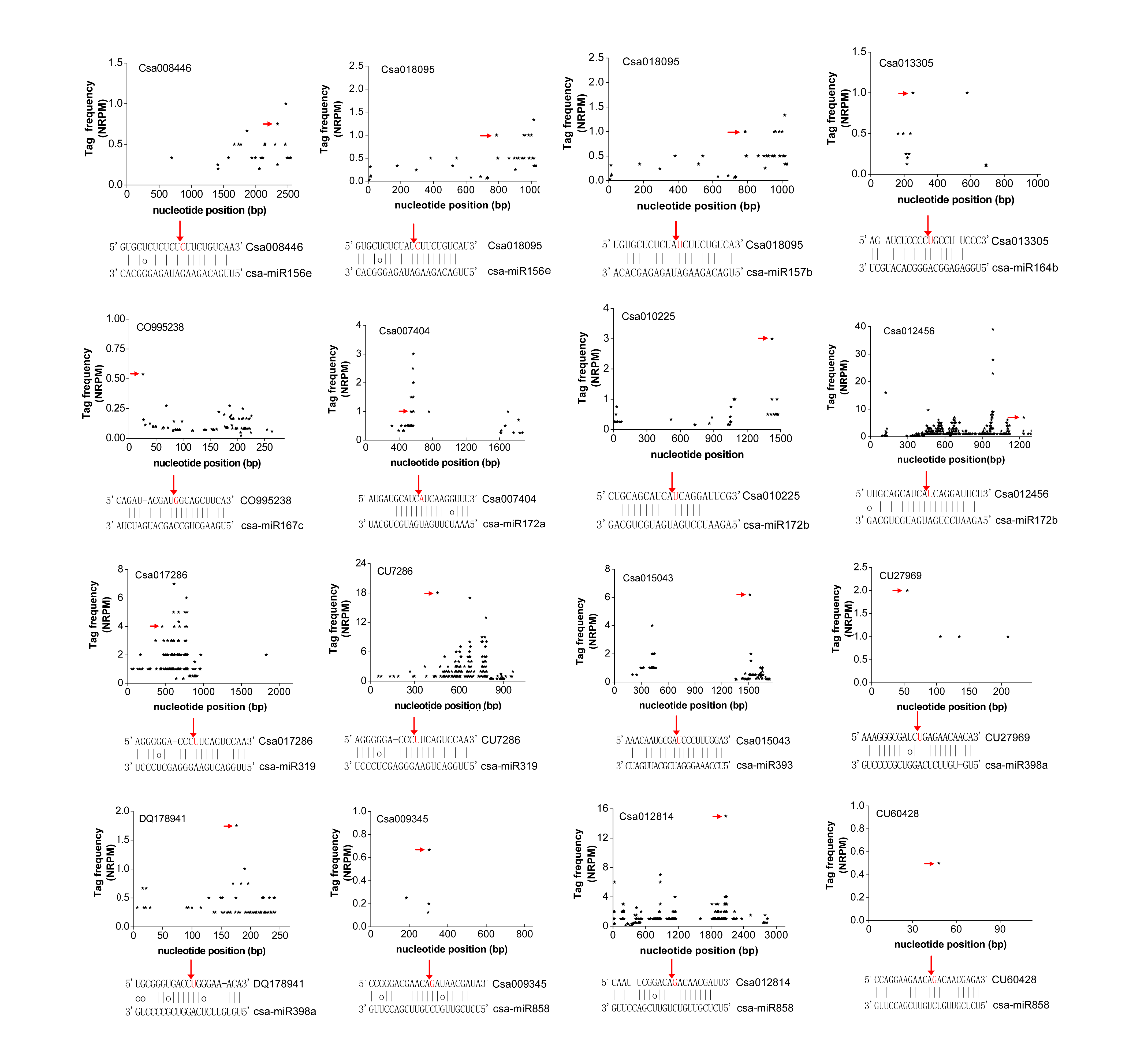

Supplement: Figure S1 — Target plots (t-plots) of miRNA targets confirmed by degradome sequencing. Signature abundance is plotted as the length of the transcript. The miRNA-directed cleavage signature is shown as the red arrow. The red letter in miRNA:mRNA alignments indicates the cleavage site detected in the degradome. (TIF) [file pone.0033040.s001.tif]
