# Supplementary material for: A Combined Approach of High-Throughput Sequencing and Degradome Analysis Reveals Tissue Specific Expression of MicroRNAs and Their Targets in Cucumber
Source: PLoS One. 2012 Mar 30;7(3):e33040. doi: 10.1371/journal.pone.0033040 (PMC3316546; doi:10.1371/journal.pone.0033040)
Supplement: Table S1 — Known miRNAs identified in cucumber and their sequence similarity to known miRNAs from other plant species. (DOC) [file pone.0033040.s002.doc]

**Table S1. Known miRNAs identified in cucumber and their sequences similarity to known miRNAs from other plant species.**

| **Family** | **miRNA name** | **miRNA seq** | **miRNA length** | **miR_ miRNA reference** | **Conserved in other plants** | | | |
| --- | --- | --- | --- | --- | --- | --- | --- | --- |
|  |  |  |  |  | **ath** | **aly** | **ptc** | **osa** |
| csa-miR156 | csa-miR156a | GCTCACTTCTCTCTCTGTCAGA | 22 | zma-miR156a* |  | ++ |  |  |
|  | csa-miR156b | GCTCACTTCTCTTTCTGTCAGT | 22 | zma-miR156d* |  | +++ |  |  |
|  | csa-miR156c | TGACAGAAGAGAGGGAGCAC | 20 | ptc-miR156k | +++ | +++ | ++++ | +++ |
|  | csa-miR156d | TGCCAGAAGAGAGTGAGCAC | 20 | ath-miR156a | +++ | +++ | +++ | +++ |
|  | csa-miR156e | TTGACAGAAGATAGAGGGCAC | 21 | mtr-miR156g | +++ | +++ | +++ | + |
| csa-miR157 | csa-miR157a | GCTCTCTATGCTTCTGTCATC | 21 | aly-miR157d* |  | ++++ |  |  |
|  | csa-miR157b | TGACAGAAGATAGAGAGCACA | 21 | ath-miR157a | +++ | +++ | +++ | +++ |
| csa-miR159 | csa-miR159a | AGCTGCTAAGCTATGGATCCC | 21 | gma-miR159d |  |  |  |  |
|  | csa-miR159b | TTTGGATTGAAGGGAGCTCTA | 21 | ath-miR159a | ++++ | ++++ | ++++ | +++ |
|  | csa-miR159c | TTTGGATTGAAGGGAGCTCT | 20 | ath-miR159a | +++ | +++ | +++ | +++ |
|  | csa-miR159d | TTTGGATTGAAGGGAGTTCT | 20 | ath-miR159a | ++ | ++ | ++ | ++ |
| csa-miR160 | csa-miR160a | GCGTATGAGGAGCCATGCATA | 21 | aly-miR160a* |  | ++++ |  |  |
|  | csa-miR160b | TGCCTGGCTCCCTGTATGCCA | 21 | ath-miR160a | ++++ | ++++ | ++++ | ++++ |
| csa-miR162 | csa-miR162a | GGAGGCAGCGGTTCATCGACC | 21 | aly-miR162a* |  | +++ |  |  |
|  | csa-miR162b | TCGATAAACCTCTGCATCCAG | 21 | ath-miR162a | ++++ | ++++ | ++++ | ++++ |
| csa-miR164 | csa-miR164a | TGGAGAAGCAGGGCACGTGCA | 21 | ath-miR164a | ++++ | ++++ | ++++ | ++++ |
|  | csa-miR164b | TGGAGAGGCAGGGCACATGCT | 21 | ptc-miR164f | + | + | +++ | ++ |
| csa-miR166 | csa-miR166 | TCGGACCAGGCTTCATTCTCG | 21 | crt-miR166a |  | ++ | ++ |  |

The miRNA name with underline denote miRNA whose precursor have been identified in cucumber genome. The abbreviations represent: ath, *Arabidopsis thaliana*; aly, *Arabidopsis lyrata*; ptc, *Populus trichocarpa*; osa, *Oryza sativa*. The plus symbols indicate: ++++, miRNA sequences of cucumber were exactly identical to those in other species; +++, miRNA sequences of cucumber were conserved in other plant species but have variations at 1 nucleotide positions; ++, miRNA sequences of cucumber were conserved in other plant species but have variations at 2 nucleotide positions; +, miRNA sequences of cucumber were conserved in other plant species but have variations at 3 nucleotide positions.

**Table S1. Continued.**

| **Family** | **miRNA name** | **miRNA seq** | **miRNA length** | **miR_ miRNA reference** | **Conserved in other plants** | | | |
| --- | --- | --- | --- | --- | --- | --- | --- | --- |
|  |  |  |  |  | **ath** | **aly** | **ptc** | **osa** |
| csa-miR167 | csa-miR167a | GGTCATGCTCTGACAGCCTCACT | 23 | aly-miR167b* |  | ++++ |  |  |
|  | csa-miR167b | TCAAGCTGCCAGCATGATCTAA | 22 | aqc-miR167 | ++ | ++ | ++ | ++ |
|  | csa-miR167c | TGAAGCTGCCAGCATGATCTA | 21 | ath-miR167a | ++++ | ++++ | ++++ | ++++ |
|  | csa-miR167d | TGAAGCTGCCAGCATGATCTG | 21 | ath-miR167d | +++ | +++ | ++++ | ++++ |
| csa-miR168 | csa-miR168 | TCGCTTGGTGCAGGTCGGGAA | 21 | ath-miR168a | ++++ | ++++ | ++++ | ++ |
| csa-miR169 | csa-miR169a | AAGCCAAGGATGAATTGCCAG | 21 | osa-miR169d | + | + | ++ | ++ |
|  | csa-miR169b | AAGCCAAGGATGAATTGCCGG | 21 | osa-miR169d | ++ | ++ | ++ | +++ |
|  | csa-miR169c | TAGCCAAAGATGACTTGCCTGT | 22 | aly-miR169n | ++ | +++ | ++ | ++ |
|  | csa-miR169d | TGAGCCAAGAATGACTTGCCGGC | 23 | ptc-miR169t | + | + | ++ | + |
|  | csa-miR169e | TGAGCCAAGGATGACTTGCCT | 21 | ath-miR169d | +++ | +++ | +++ | + |
| csa-miR170 | csa-miR170 | TATTGGCCCGGTTCACTCAGA | 21 | aly-miR170* |  | +++ |  |  |
| csa-miR171 | csa-miR171 | TTGAGCCGTGCCAATATCACG | 21 | ath-miR171b | ++++ | ++++ | ++++ | + |
| csa-miR172 | csa-miR172a | AAATCTTGATGATGCTGCAT | 20 | ath-miR172a | ++ | ++ | ++ | ++ |
|  | csa-miR172b | AGAATCCTGATGATGCTGCAG | 21 | mtr-miR172 | +++ | +++ | +++ | ++ |
|  | csa-miR172c | GAGAATCTTGATGATGCTGCA | 21 | ath-miR172a | +++ | +++ | +++ | +++ |
|  | csa-miR172d | GTAGCATTATCAAGATTCACA | 21 | aly-miR172a* |  | ++ |  |  |
| csa-miR319 | csa-miR319 | TTGGACTGAAGGGAGCTCCCT | 21 | ath-miR319a | ++++ | ++++ | +++ | ++ |
| csa-miR390 | csa-miR390a | AAGCTCAGGAGGGATAGCGCC | 21 | ath-miR390a | ++++ | ++++ | ++++ | ++++ |
|  | csa-miR390b | CGCTATCCATCCTGAGTTTCC | 21 | aly-miR390a* |  | +++ |  |  |
| csa-miR393 | csa-miR393-3p | TCATGCTATCCCTTCGGATT | 20 | aly-miR393a* |  | + |  |  |
|  | csa-miR393 | TCCAAAGGGATCGCATTGATC | 21 | ath-miR393a | +++ | ++++ | ++++ | ++++ |
| csa-miR394 | csa-miR394 | TTGGCATTCTGTCCACCTCC | 20 | ath-miR394a | ++++ | ++++ | ++++ | ++++ |
| csa-miR396 | csa-miR396a | CCACAGCTTTCTTGAACTGCA | 21 | ath-miR396a | ++ | ++ | ++ | ++ |
|  | csa-miR396b | GTTCAAGAAAGCTGTGGGAGA | 21 | zma-miR396g* | + | ++ | + | + |
|  | csa-miR396c | GTTCAATAAAGCTGTGGGAAG | 21 | aly-miR396a* |  | ++++ |  |  |
|  | csa-miR396d | TTCCACAGCTTTCTTGAACTT | 21 | ath-miR396b | ++++ | ++++ | ++++ | ++++ |

**Table S1. Continued.**

| **Family** | **miRNA name** | **miRNA seq** | **miRNA length** | **miR_ miRNA reference** | **Conserved in other plants** | | | |
| --- | --- | --- | --- | --- | --- | --- | --- | --- |
|  |  |  |  |  | **ath** | **aly** | **ptc** | **osa** |
| csa-miR397 | csa-miR397 | TCATTGAGTGCAGCGTTGATG | 21 | ath-miR397a | ++++ | ++++ | ++++ | ++++ |
| csa-miR398 | csa-miR398a | TGTGTTCTCAGGTCGCCCCTG | 21 | osa-miR398b | +++ | +++ | ++++ | ++++ |
|  | csa-miR398b | TTGTGTTCTCAGGTCACCCCT | 21 | ath-mIR398a | +++ | +++ | +++ | +++ |
| csa-miR399 | csa-miR399a | AGGGCTTCTCTCCATTGGCAGG | 22 | aly-miR399b* |  | + |  |  |
|  | csa-miR399b | TGCCAAAAGAGACTTGCCC | 19 | osa-miR399h |  |  |  | + |
|  | csa-miR399c | TGCCAAAGGAGAGTTGCCCTT | 21 | ath-miR399b | +++ | +++ | +++ | +++ |
|  | csa-miR399d | TGCCAAAGGAGATTTGCCCGG | 21 | ath-miR399f | ++++ | ++++ | ++++ | +++ |
| csa-miR408 | csa-miR408a | ATGCACTGCCTCTTCCCTGGC | 21 | ath-miR408 | ++++ | ++++ | ++++ | +++ |
|  | csa-miR408b | CGGGGAACAGACAGAGCATG | 20 | aly-miR408* |  | + |  |  |
| csa-miR477 | csa-miR477 | TTCTCTCCCTCAAGGGCTTCGA | 22 | aqc-miR477e |  |  |  |  |
| csa-miR530 | csa-miR530 | TGCATTTGCACCTACACCTTC | 21 | ptc-miR530a |  |  | ++ | + |
| csa-miR827 | csa-miR827 | TTAGATGACCATCAACGAACG | 21 | aly-miR827 | ++ | +++ | ++ | + |
| csa-miR858 | csa-miR858 | TCTCGTTGTCTGTTCGACCTTG | 22 | ath-miR858 | ++ | ++ |  |  |
| csa-miR1515 | csa-miR1515 | TCATTTTTGCGTGCAATGATCC | 22 | csi-miR1515 |  |  |  |  |
| csa-miR2111 | csa-miR2111 | TAATCTGCATCCTGAGGTTTA | 21 | ath-miR2111a | ++++ | ++++ |  |  |
| csa-miR2950 | csa-miR2950 | TTCCATCTCTTGCACACTGGA | 21 | vvi-miR2950 |  |  |  |  |
