# Supplementary material for: A Combined Approach of High-Throughput Sequencing and Degradome Analysis Reveals Tissue Specific Expression of MicroRNAs and Their Targets in Cucumber
Source: PLoS One. 2012 Mar 30;7(3):e33040. doi: 10.1371/journal.pone.0033040 (PMC3316546; doi:10.1371/journal.pone.0033040)
Supplement: Table S2 — Primer sequences used for qRT-PCR. (DOC) [file pone.0033040.s003.doc]

**Table S2. Primer sequences used for qRT-PCR.**

| **Name** | **primers (5’ → 3’)** |
| --- | --- |
| csa-miR156 | TGACAGAAGAGAGGGAGCAC |
| csa-miR159 | TTTGGATTGAAGGGAGCTCTA |
| csa-miR160 | TGCCTGGCTCCCTGTATGCCA |
| csa-miR164 | TGGAGAAGCAGGGCACGTGCA |
| csa-miR166 | TCGGACCAGGCTTCATTCTCG |
| csa-miR171 | TTGAGCCGTGCCAATATCACG |
| csa-miR397 | TCATTGAGTGCAGCGTTGATG |
| csa-miR398 | TGTGTTCTCAGGTCGCCCCTG |
| csa-miR408 | ATGCACTGCCTCTTCCCTGGC |
| csa-miR477 | TTCTCTCCCTCAAGGGCTTCGA |
| csa-miR530 | TGCATTTGCACCTACACCTTC |
| csa-miR827 | TTAGATGACCATCAACGAACG |
| csa-miR858 | TCTCGTTGTCTGTTCGACCTTG |
| csa-miRn1-3p | AGGTGTCATCTCACTGCGGTA |
| csa-miRn2-5p | TGCTGCTCATTCGTTAGTTCA |
| U6-F | GGGGACATCCGATAAAATT |
| U6-R | TGTGCGTGTCATCCTTGC |
